# Functional disruption of oxytocin projections participates atypical social and anxiety-like behaviours in BTBR mouse model of autism

**DOI:** 10.1098/rsob.240387

**Published:** 2025-08-27

**Authors:** Yuki Higuchi, Akihiko Ozawa, Ryuki Kobayashi, Toshihiro Konno, Hiroyuki Arakawa

**Affiliations:** ^1^ Department of Systems Physiology, University of the Ryukyus School of Medicine, Nakagami-gun, Japan; ^2^ Department of Biomedical Science, Florida Atlantic University, Boca Raton, FL, USA; ^3^ Subtropical Agro-Environmental Sciences, University of the Ryukyus - Uehara Campus, Nakagami District, Okinawa Prefecture, Japan; ^4^ Department of Pharmacology, University of Michigan Medical school, Ann Arbor, MI, USA

**Keywords:** oxytocin, neural circuits, autism, social behaviour, anxiety, chemogenetics

## Abstract

Oxytocin (OXT) neurons in the paraventricular nucleus of the hypothalamus (PVN), which send projections to the medial amygdala (MeA) and the bed nucleus of the stria terminalis (BnST), are implicated in regulation of prosocial-emotional behaviours and abnormalities resembling autism spectrum disorders (ASD). Compared with standard C57BL6J (B6) mice, BTBR mice, a behaviour-based ASD model, exhibited decreased densities of OXT^PVN^ neurons and attenuated OXT neuronal responses to a social encounter. OXT receptor mRNA expressions in the MeA and BnST as a response to a social encounter were blunted in BTBR mice. OXT promoter retrograde viral tracing revealed that the OXT^PVN→BnST^ projections were defective in those BTBR mice. Thus, chemogenetic excitation of OXT^PVN→MeA^ neurons using OXT promoter adeno-associated viruses (AAV) enhanced anxiety-like behaviour and facilitated social investigation in both strains, while excitation of OXT^PVN→BnST^ neurons attenuated anxiety-like behaviour along with social investigation in B6 mice and failed to induce a change in their socio-emotional behaviours in BTBR mice. Altogether, OXT circuits serve as a key regulator for socio-emotional behaviour; MeA-OXT projection facilitates social investigation and anxiety-like behaviour, while BnST-OXT projection conversely attenuates these behaviours; hence a defect of the OXT^PVN→BnST^ circuits contributes to the development of ASD-like social phenotypes in BTBR mice.

**Figure:**
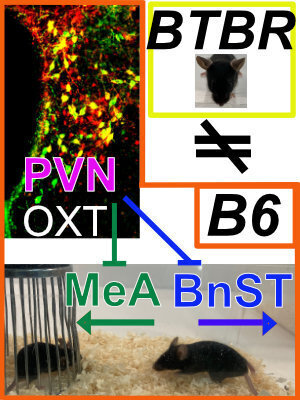

## 1. Introduction

Social interaction is a fundamental behaviour in all animal species for sustaining adaptive life and well-being. Engaging in positive social (i.e. prosocial) interactions robustly benefits health across the lifespan [1–3]. Conversely, the disruption of prosocial relationships has emerged as a major symptom in many psychiatric diseases and neurodegenerative diseases [4–7]. Oxytocin (OXT) is a neuropeptide involved in a key mechanism that regulates prosocial behaviour, as evidenced in several different species, including humans [8–10]. Central OXT synthesis occurs in the paraventricular nucleus (PVN) and the supraoptic nucleus (SON) of the hypothalamus as well as in the accessory magnocellular nuclei of the hypothalamus, such as strio- and septo-hypothalamic nuclei [10,11]. The central OXT is mainly transported via axonal projections from the PVN, distributed to various brain regions, including the basal ganglia area, such as nucleus accumbens, septal nucleus, bed nucleus of the stria terminalis (BnST), amygdala subregions including medial amygdala (MeA), and paraventricular thalamic nucleus, and mid brain areas including the raphe nucleus and periaqueductal grey [12–14]. In particular, the OXT^PVN-->MeA^ neurons are required for the promoting social investigation (e.g. approach and contacts) [15–17], while those OXT^PVN-->BnST^ neurons contribute to activating social avoidance and defensive behaviour by circuit-specific OXT action [18,19].

The PVN is a complex, multifunctional and multitransmitter nucleus, which modulates many central processes, including stress-related hormonal regulation [20]. Activation of the PVN neurons follows OXT release and OXT-ergic circuit-specific processing as well as other peptide releases such as arginine-vasopressin (AVP) and corticotrophin-releasing hormone (CRH) [20,21]. AVP, an OXT homologue peptide, also plays a discrete role in the regulation of sexual-dimorphic social behaviours [9,11]. Abnormalities in either the OXT peptide or its receptor have been associated with social deficits that are prevalent in many psychiatric and neurodevelopmental disorders, including autism spectrum disorders (ASDs) [22,23]. While whole-brain (e.g. intranasal) treatment with OXT incoherently failed to affect prosocial behaviour in mice [24] and alleviate ASD symptoms in clinical trials [25], circuit-specific processing of OXT via the complex PVN along with the SON network underlies for appropriate executions of prosocial behaviour.

**Figure 1.**
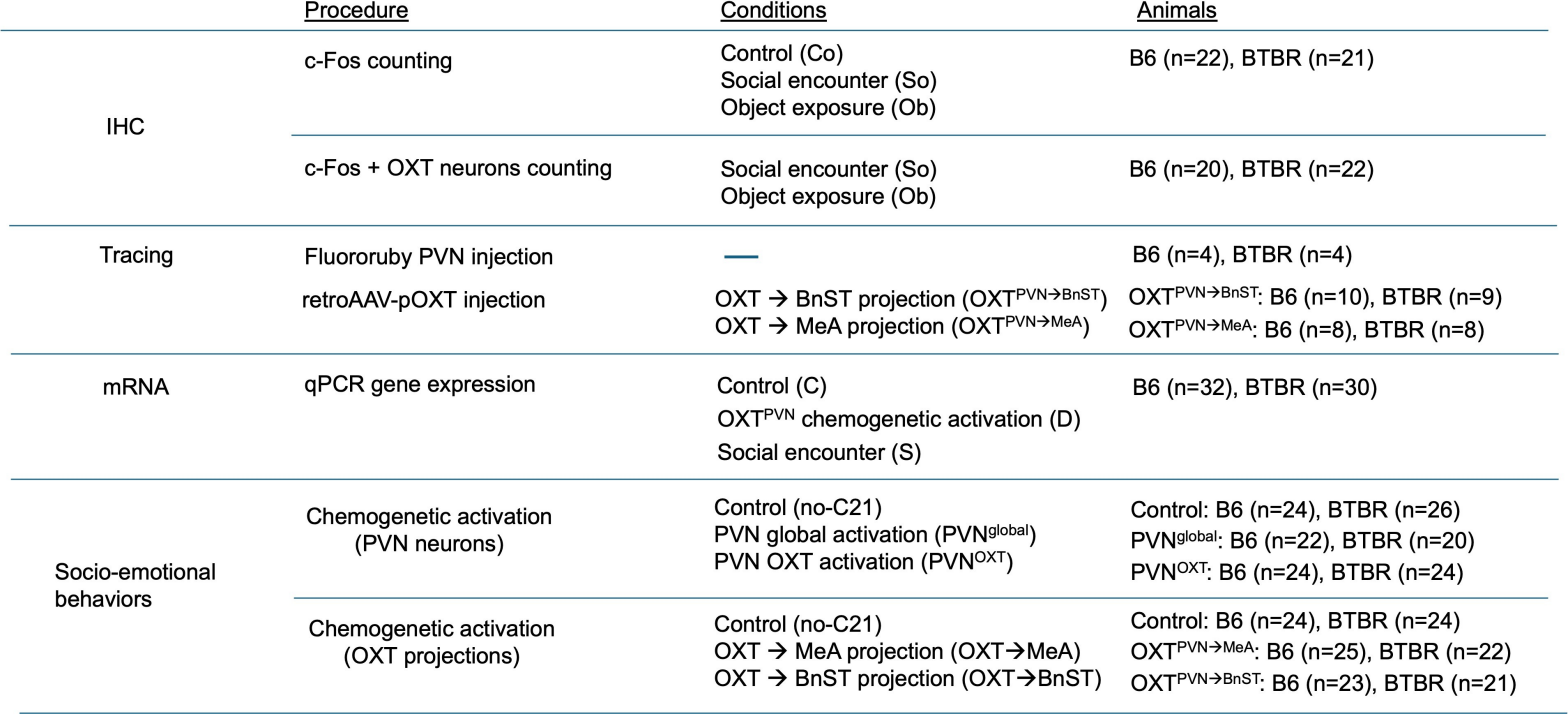
Experimental design and applied procedures for analysing OXT circuit function in B6 and BTBR mice. Immunohistochemistry (IHC) was used for c-Fos+cells counting and c-Fos+cells and OXT+cells co-expressions. Neural tracing was conducted with an anterograde tracer, Fluoro-Ruby, injected into the PVN of B6 and BTBR mice and retrograde AAV with OXT promoter injected into the BnST(OXT^PVN→BnST^) or MeA (OXT^PVN→MeA^). mRNA expression analysis using qPCR was performed in several brain regions of B6 and BTBR mice; they were confronted with a same-sex, unfamiliar mouse (social encounter) or received bilateral AAV (AAV8-pOXT-hM3Dq) injection in the PVN (OXT^PVN^) and a DREADD ligand, C21 injection. Behavioural assessments with chemogenetic excitation of targeted cells and regions were performed: groups included control (AAV injected but no-C21 treated), PVN^global^ (non-specific hM3Dq in PVN), OXT^PVN^ (OXT promoter hM3Dq in PVN), OXT^PVN→MeA^ (retroAAV OXT promoter hM3Dq in MeA) and OXT^PVN→BnST^ (retroAAV OXT promoter hM3Dq in BnST).

Mice are a highly social species that provide an exceptional tool for elucidating the neural mechanisms underlying prosocial behaviour and its deficits [26]. Multiple lines of evidence have indicated that BTBR T+Itpr3 tf/J (BTBR) mice serve as a behaviour-based ASD model resembling the characteristics of human ASD, including impaired prosocial interaction and persistent, repetitive behaviours [27,28]. A decreased neural activation of the PVN in BTBR mice in response to the presence of social stimuli compared with that in a control mouse strain (e.g. C57BL/6J; B6 mice) has been documented [29–31]. Altered OXT neuronal morphology in the PVN was also evident in both sexes of BTBR mice compared with B6 mice [29,31]. We hypothesized that (i) OXT neuronal signalling in the PVN is relayed via circuit-specific processes to control appropriate execution of prosocial behaviour, and that (ii) malformation or disconnection of these OXT circuits underlies social deficits exhibited by BTBR mice. Using immunohistochemical mapping of neural activity patterns with c-Fos and OXT neurons and cell-type specific tracing of OXT projections by using the OXT promoter, AAV, we identify altered OXT activity and OXT projecting patterns in the PVN of BTBR mice. Our data obtained by chemogenetic manipulation of PVN neurons demonstrate that two massive OXT projections, OXT^PVN→MeA^ and OXT^PVN→BnST^, discretely regulate social and non-social behaviours, and a modification of these projections and binding receptor expressions contribute to social deficit phenotypes in BTBR mice (figure 1). Therefore, the data demonstrate a circuitry role for PVN OXT signalling in the expression of prosocial and anxiety-related behaviour and reveal a possible OXT mechanism underlying behavioural deficiency in BTBR mice as an ASD mouse model.

## 2. Results

### 2.1. Paraventricular nucleus neuronal and oxytocin activity in response to social and non-social encounters

**Figure 2.**
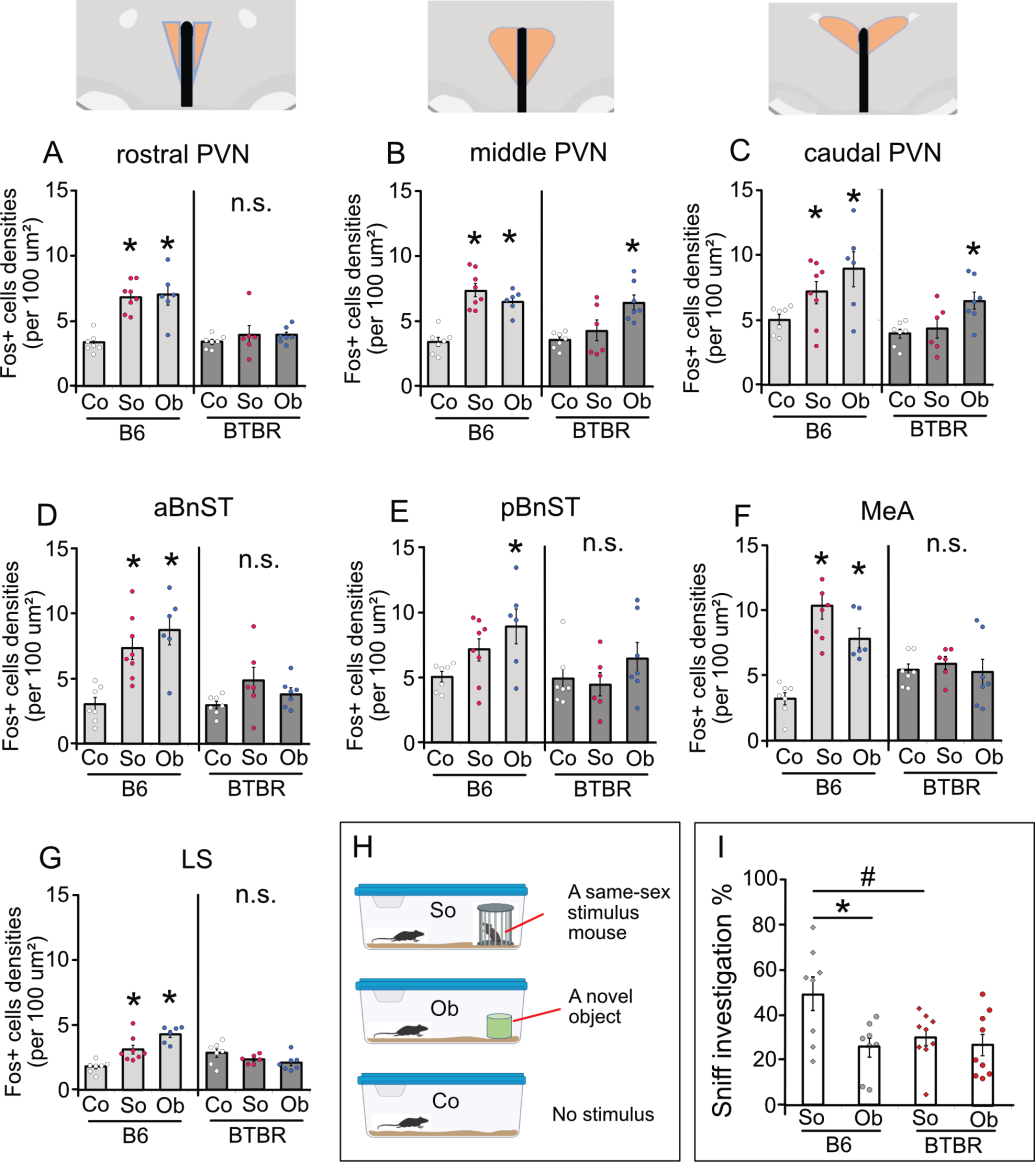
Neural activity marker, c-Fos expression of B6 and BTBR mice in PVN and related brain regions, in response to exposure to an object (Ob) or social (So) stimulus. Brain regions analysed included the rostral (A), middle (B), and caudal (C) PVN portions, anterior BnST (D), and posterior BnST (E), medial amygdala (MeA) (F), and lateral septum (LS)(G). (H) Test conditions of stimulus exposure. (I) Sniff investigation of B6 and BTBR mice exposed to a social (So) or object (Ob) stimulus. * Indicates differences between stimuli compared with homecage controls (Co), *p* < 0.05. # Indicates difference between strain, *p* < 0.05. n.s., Not significant.

We firstly confirmed neural responses of the PVN regions to social (i.e. a different strain mouse) and non-social (i.e. an object) stimuli in B6 and BTBR mice. To map neural activity patterns in the PVN, we performed immunohistochemistry for c-Fos across the rostral–caudal divides of the PVN (figure 2A,C) and its projecting regions; MeA (figure 2F), BnST (figure 2D,E) and lateral septum (LS) (figure 2G) in B6 and BTBR mice that were exposed to a social (a novel same-sex mouse) or non-animated object, or remained in a home cage as a no exposure control (figure 2H). For B6 mice, significantly more cells expressing c-Fos in response to either social or non-social stimuli compared with the control condition were documented across the rostral to caudal portions of the PVN (rostral PVN: *F*(2,18) = 18.18, *p* < 0.0001, middle PVN: *F*(2,18) = 25.13, *p* < 0.0001, and caudal PVN: *F*(2,18)= 20.94, *p* = 0.0004) (figure 2A–C). For BTBR mice, however, the induction of c-Fos was found through the middle to caudal portions of the PVN only by an object, but not social, exposure (rostral PVN: *F*(2,17) = 0.58, *p* = 0.568, middle PVN: *F*(2,17) = 7.69, *p* < 0.0042, and caudal PVN: *F*(2,17)= 5.58, *p* = 0.014). This c-Fos induction in response to an object exposure was not evident in the rostral portion of the PVN. In the projecting regions from the PVN, the numbers of c-Fos+cells were increased both in response to social and non-social (object) exposures in aBnST (*F*(2,18) = 11.73, *p* = 0.0005), MeA (*F*(2,18) = 21.01, *p* < 0.0001) and LS of B6 mice (*F*(2,18) = 18.79, *p* < 0.0001). An object exposure also increased the number of c-Fos+cells in pBnST of B6 mice (*F*(2,18) = 11.73, *p* = 0.0005). These c-Fos responses to either stimulus were blunted in BTBR mice across brain regions including aBnST (*F*(2,17) = 2.08, *p* = 0.156), pBnST (*F*(2,17) = 1.26, *p* = 0.31), MeA (*F*(2,17) = 0.27, *p* = 0.269) and LS (*F*(2,17) = 2.40, *p* = 0.121). Accordingly, sniffing/contact investigation towards exposed stimuli was demonstrated in both B6 and BTBR mice; however, these were attenuated in BTBR mice when confronted with a social stimulus (strain: *F*(1,30) = 3.12, *p* = 0.088, stimulus: *F*(1,30) = 7.02, *p* = 0.013, and interaction: *F*(1,30) = 3.98, *p* = 0.046) (figure 2I). Therefore, a blunted c-Fos induction in targeted regions in response to social encounter exhibited by BTBR mice is covariant with decreased social investigation.

**Figure 3.**
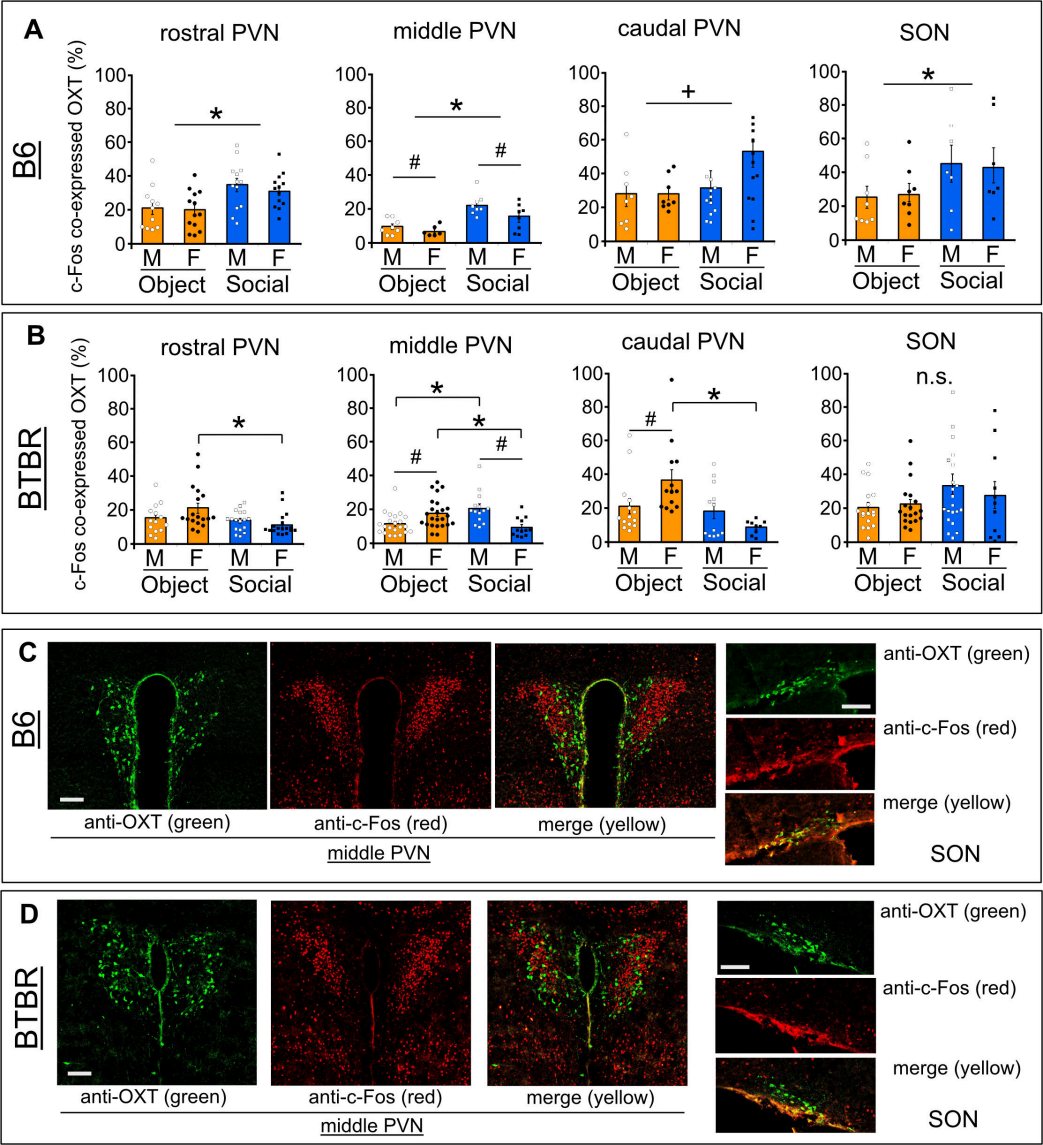
Ratio of OXT+cell co-expressions with c-Fos+cells in the PVN subportions and SON of B6 and BTBR mice. Across the rostral, middle, to caudal PVN regions and SON of B6 (A) and BTBR (B) mice, c-Fos co-expression ratio of OXT neurons (i.e., activated OXT neurons) in response to a social encounter or object exposure. * Indicates difference between exposed stimuli, *p* < 0.05, +*p*< 0.10. # Indicates sex difference, *p* < 0.05. n.s., Not significant. A representation of PVN and SON fluorescence-expression of OXT+ (green), c-Fos+ (red), and merged (yellow) in B6 (C) and BTBR (D) mice. Scale bar = 100 μm.

We also double-stained OXT+neurons and c-Fos+neurons in PVN sub-portions and SON of B6 and BTBR mice (figure 3). Co-expressions of OXT+neurons with c-Fos+neurons indicate activated OXT neurons in response to either an object or social exposure (figure 3C,D). For B6 mouse brain (figure 3A), the ratio of c-Fos co-expressed OXT+neurons was greater in social confrontation than those in object confrontation in both sexes of B6 mice at the rostral PVN (stimulus: *F*(1,46)= 11.13, *p* = 0.0017, sex: *F*(1,46) = 0.84, *p* = 0.365, interaction: *F*(1,46) = 0.34, *p* = 0.564), middle PVN (stimulus: *F*(1,26)= 22.82, *p* = 0.0001, sex: *F*(1,26) = 5.32, *p* = 0.029, interaction: *F*(1,26) = 1.33, *p* = 0.26), and also at SON (stimulus: *F*(1,26)= 4.575, *p* = 0.042, sex: *F*(1,26) = 0.012, *p* = 0.915, interaction: *F*(1,26) = 0.49, *p* = 0.827). This trend was also found at the caudal PVN of B6 (stimulus: *F*(1,38)= 3.13, *p* = 0.085, sex: *F*(1,38) = 1.44, *p* = 0.238, interaction: *F*(1,38) = 1.21, *p* = 0.279). Conversely, BTBR mice did not show consistent tendencies in co-expression of OXT+neurons with c-Fos across the PVN and SON regions (figure 3B). A greater co-expression ratio was found in the rostral PVN (*p* = 0.0088) and caudal PVN (*p* = 0.0015) of female BTBR mice in an object confrontation compared with social confrontation (rostral PVN, stimulus: *F*(1,63)= 5.82, *p* = 0.019, sex: *F*(1,63) = 0.85, *p* = 0.36, interaction: *F*(1,63) = 3.68, *p* = 0.06 and caudal PVN, stimulus: *F*(1,44)= 9.81, *p* = 0.003, sex: *F*(1,44) = 0.37, *p* = 0.545, interaction: *F*(1,44) = 6.57, *p* = 0.014). In the middle PVN, a co-expression ratio was greater in males confronted with a social stimulus than those confronted with an object (*p* = 0.0018), while a higher co-expression ratio was found in females when confronted with an object compared with those confronted with a social stimulus (*p* = 0.0052) (stimulus: *F*(1,68)= 0.07, *p* = 0.79, sex: *F*(1,68) = 1.78, *p* = 0.187, interaction: *F*(1,68) = 20.81, *p* < 0.0001). In addition, there was no significant difference between exposed stimuli in the co-expression ratio of the SON (stimulus: *F*(1,65)= 3.40, *p* = 0.07, sex: *F*(1,65) = 0.21, *p* = 0.65, interaction: *F*(1,65) = 0.58, *p* = 0.45).

### 2.2. mRNA profiles following chemogenetic oxytocin activation and social encounter

**Figure 4.**
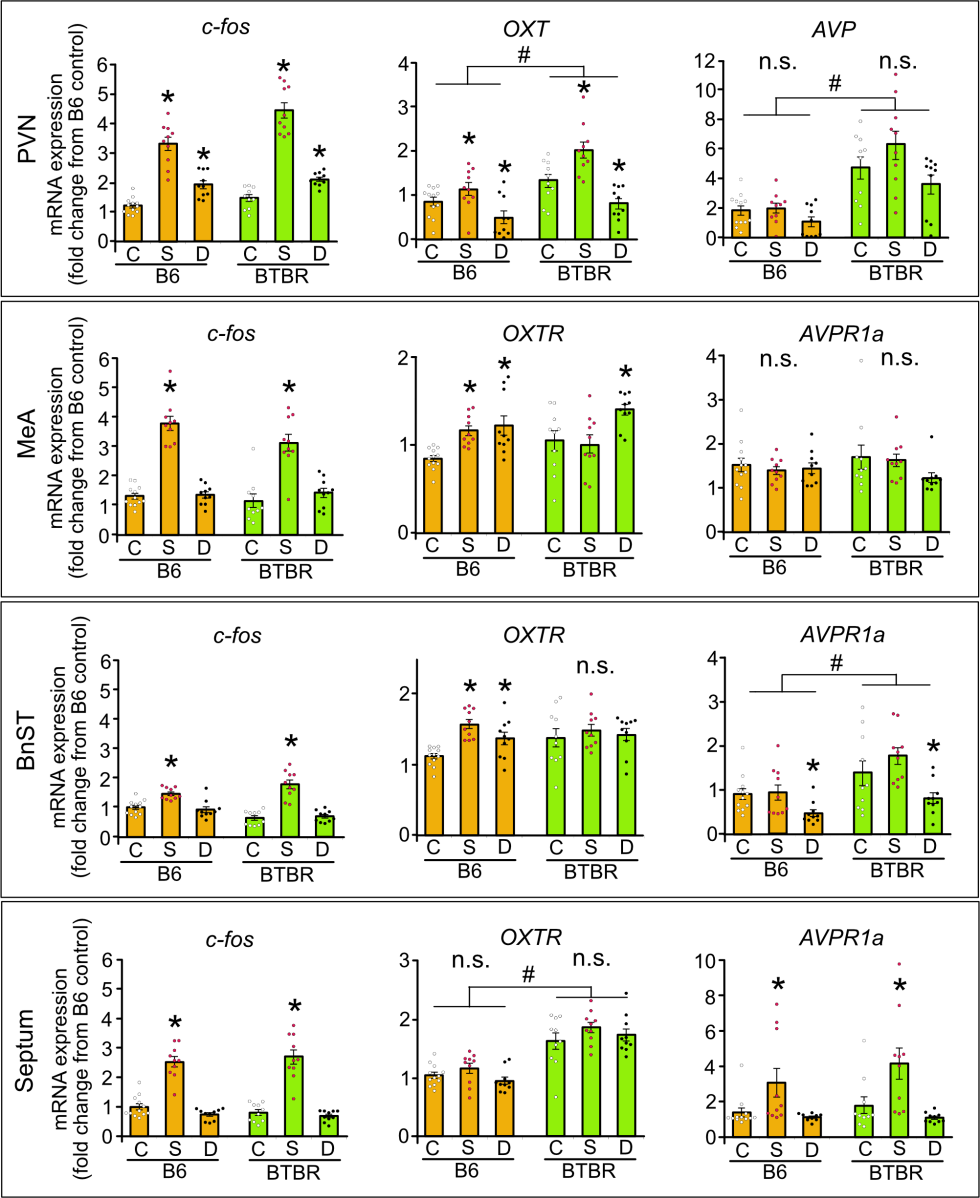
The mRNA expression of *c-fos***,** *OXT***,** *AVP* and its homologue receptors (*OXTR* and *AVPR1a*) in the PVN and its projecting regions induced by social encounter (S) or chemogenetic activation of OXT^PVN^ neurons (D) in B6 and BTBR mice. In the PVN, *c-fos*, *OXT* and AVP mRNA expressions were calculated. In the MeA, BnST and septum, *c-fos, OXTR,* and *AVPR1a* mRNA were assessed. These mice were exposed to a social stimulus (S) or received a chemogenetic activation of OXT^PVN^ neurons and a DREADD ligand treatment (D), or control vehicle treatment (C). * Difference between conditions compared with controls, # difference between strains, *p* < 0.05. n.s., Not significant.

While OXT is known to play a modulatory role in anxiety behaviour [32], stress responses [20,33] and social behaviour [15,16], the effects of exogenous OXT are still variable [34] and the brain regions that transmit OXT signals from the PVN for proper execution of behaviour have not fully been identified. We applied chemogenetic manipulation on the OXT^PVN^ neurons via AAV using the selective OXT promoter transducing hM3Dq to determine the OXT cell-type specificity of the PVN reactions. To validate OXT cell reaction patterns, we conducted quantitative polymerase chain reaction (qPCR) analyses on selected brain regions of B6 and BTBR mice exposed to either a social stimulus (S; a novel same-sex mouse) or a chemogenetic excitation of OXT^PVN^ neurons (D) without a social stimulus (figure 4). We analysed gene expressions that are relevant to OXT neuronal activities via the PVN circuit, including *c-fos* (as a neural activity marker), *OXT*, OXT receptors (*OXTR*), vasopressin (*AVP*) and vasopressin 1 a receptors (*AVPR1a*) in the PVN and its projecting regions, MeA, BnST and septum.

In both B6 and BTBR mice, an increase in *c-fos* mRNA expression was observed in all selected regions following a social encounter, but only in the PVN by chemogenetic OXT^PVN^ activation, where AAV8-pOXT-hM3Dq was injected (PVN; strain: *F*(1,56) = 15.62, *p* = 0.0002, stimulus: *F*(2,56) = 135.23, *p* < 0.0001, interaction: *F*(2,56) = 5.34, *p* = 0.008; MeA; strain: *F*(1,56) = 2.45, *p* = 0.123, stimulus: *F*(2,56) = 80.85, *p* < 0.0001, interaction: *F*(2,56) = 1.80, *p* = 0.175; BnST; strain: *F*(1,56) = 0.87, *p* = 0.354, stimulus: *F*(2,56) = 53.59, *p* < 0.0001, interaction: *F*(2,56) = 8.07, *p* = 0.0008; septum; strain: *F*(1,56) = 0.006, *p* = 0.938, stimulus: *F*(2,56) = 112.67, *p* < 0.0001, interaction: *F*(2,56) = 0.84, *p* = 0.439). BTBR mice showed a higher *OXT* expression in the PVN than B6 mice. Social encounters induced *OXT* gene expression in the PVN of both strains, while chemogenetic activation decreased it in the PVN of both strains (PVN; strain: *F*(1,56) = 17.91, *p* < 0.0001, stimulus: *F*(2,56) = 14.81, *p* = 0.0003, interaction: *F*(2,56) = 0.40, *p* = 0.672). Both social encounters and chemogenetic activation induced *OXTR* mRNA expressions in the MeA and BnST of B6 mice, but only chemogenetic activation induced *OXTR* expression in MeA of BTBR mice (MeA; strain: *F*(1,56) = 1.31, *p* = 0.258, stimulus: *F*(2,56) = 9.77, *p* = 0.0002, interaction: *F*(2,56) = 2.75, *p* = 0.073). BTBR mice exhibited a higher *OXTR* gene expression in the septum than B6 mice, regardless of stimulus exposure (septum; strain: *F*(1,56) = 84.12, *p* < 0.0001, stimulus: *F*(2,56) = 2.38, *p* = 0.102, interaction: *F*(2,56) = 0.62, *p* = 0.544). BTBR mice showed a higher *AVP* expression in the PVN than B6 mice, regardless of stimulus exposure (PVN; strain: *F*(1,56) = 43.64, *p* < 0.0001, stimulus: *F*(2,56) = 2.45, *p* = 0.161, interaction: *F*(2,56) = 1.14, *p* = 0.326). In addition, BTBR mice exhibited a higher *AVPR1a* expression in the BnST than B6 mice, regardless of stimulus exposure. The social encounter had no impact on *AVPR1a* gene expressions in the MeA and BnST of both B6 and BTBR mice (MeA; strain: *F*(1,56) = 0.27, *p* = 0.609, stimulus: *F*(2,56) = 9.77, *p* = 0.0002, interaction: *F*(2,56) = 1.12, *p* = 0.334, BnST; strain: *F*(1,56) = 16.17, *p* = 0.0002, stimulus: *F*(2,56) = 8.85, *p* = 0.0005, interaction: *F*(2,56) = 1.04, *p* = 0.359), while it increased *AVPR1a* expression in the septum of both B6 and BTBR mice (septum; strain: *F*(1,56) = 1.29, *p* = 0.262, stimulus: *F*(2,56) = 8.34, *p* = 0.0007, interaction: *F*(2,56) = 0.40, *p* = 0.673). Chemogenetic OXT^PVN^ excitation had no impact on *AVPR1a* expression in the MeA or septum of both B6 and BTBR mice, while it decreased *AVPR1a* expression in the BnST of both B6 and BTBR mice.

Chemogenetic activation of OXT^PVN^ neurons downregulated *OXT* gene expressions in the PVN and *AVPR1a* expressions in the BnST of both strains. The chemogenetic OXT^PVN^ excitation also upregulated *OXTR* expressions in the MeA of both strains, while in the BnST, changes in the *OXTR* mRNA expression (upregulation) were only observed in B6 mice but not in BTBR mice, which represents only strain difference in mRNA expression pattern following achemogenetic excitation examined in the present study.

### 2.3. Oxytocin neural projection patterns reveal strain difference

**Figure 5.**
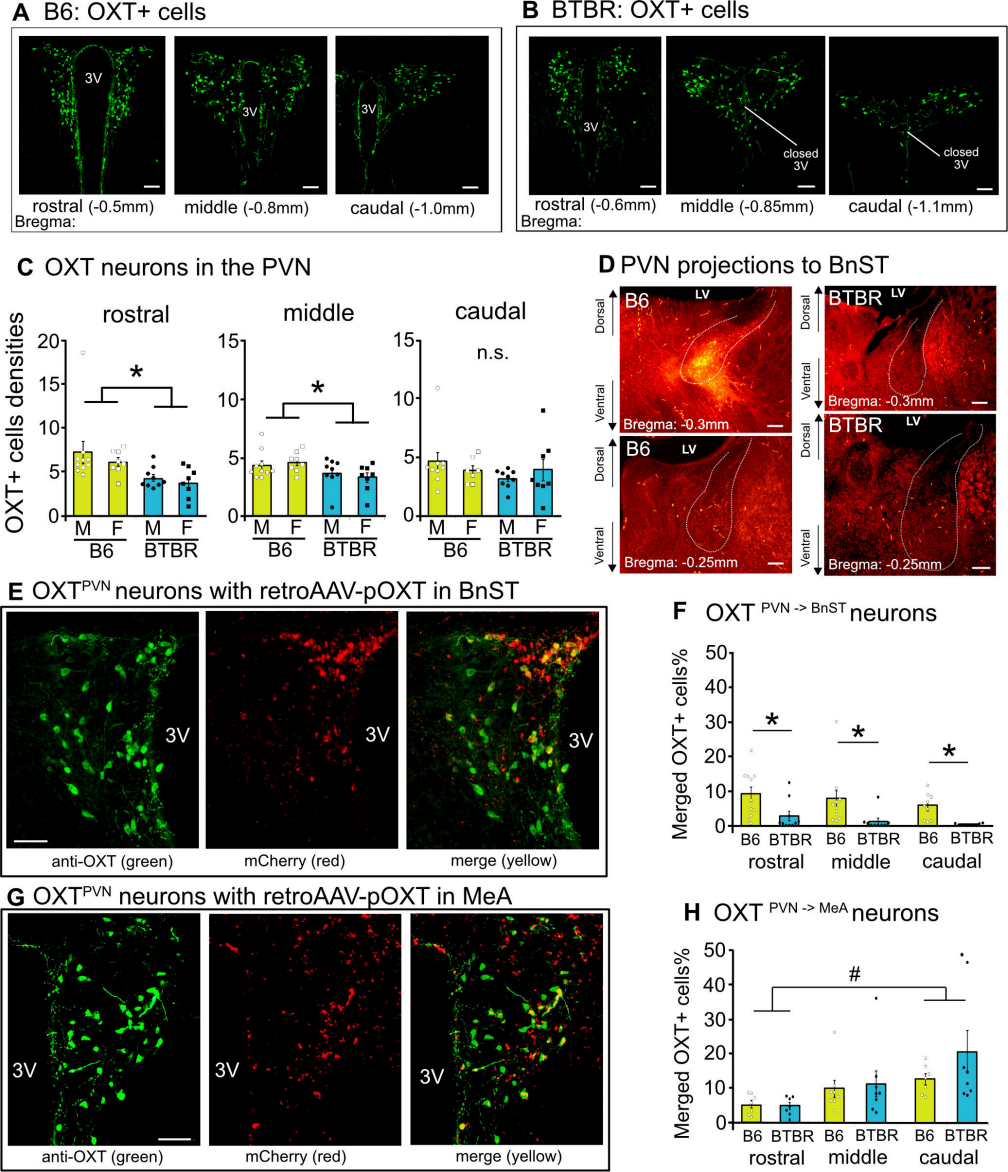
OXT+cells and projections in B6 and BTBR mice. Representative immunostaining of anti-OXT antibody (green) across the rostral to caudal PVN of B6 (A) and BTBR (B) mice. (C) The densities of OXT+cells across three PVN sub-portions in B6 and BTBR mice. (D) Representative pictures of anterograde (Fluoro-Ruby) tracing axons from the PVN to BnST in B6 and BTBR mice. (E,G) Representative pictures show OXT+cells (green), OXT promoter AAV with mCherry expressing cells (red), and those merged (yellow) cells in B6 mice that were injected with retroAAV into the BnST (OXT^PVN→BnST^ neurons; (E) or those B6 mice received retroAAV injection into the MeA (OXT^PVN→MeA^ neurons; (G). (F,H) The ratio of OXT+cells co-expressing mCherry (merged cells) across rostral to caudal PVN portions in B6 and BTBR mice that received OXT^PVN→BnST^ or OXT^PVN→MeA^ transfection. * Indicates differences between strains, *p* < 0.05. # Indicates differences between PVN portions, *p* < 0.05. n.s., Not significant. Scale bar = 100 μm. 3V: third ventricle, LV: lateral ventricle

OXT neurons of the PVN are primarily responsible for the innervation of forebrain regions [10,35]. While dendritic OXT release is also evident [36], OXT^PVN^ neurons send axonal projections to various brain regions [12,13]. We chose two massive OXT projecting regions for analysis, MeA and BnST, based on functional contribution to the regulation of social behaviour [14,15,17,18]. Firstly, immunohistochemical analysis confirmed that OXT neurons are distributed across the rostral to caudal portions of the PVN in both B6 and BTBR mice, without any sex differences (figure 5A,B). Densities of OXT neurons are lower in BTBR mice than those in B6 mice particularly in the rostral (strain: *F*(1,31) = 9.41, *p* = 0.005, sex: *F*(1,31) = 0.87, *p* = 0.358, and interaction, *F*(1,31) = 0.097, *p* = 0.758) and middle portions of the PVN (strain: *F*(1,31) = 6.42, *p* = 0.017, sex: *F*(1,31) = 0.04, *p* = 0.84, and interaction: *F*(1,31) = 0.84, *p* = 0.367) (figure 5C). There was no strain difference in the densities of OXT neurons in the caudal PVN (strain: *F*(1,31) = 0.99, *p* = 0.326, sex: *F*(1,31) = 0.0004, *p* = 0.984, interaction: *F*(1,31) = 1.14, *p* = 0.295).

Secondly, to confirm strain differences in global PVN neural projections, an anterograde tracer (Fluoro-Ruby) injected into the PVN of B6 and BTBR mice (each *n* = 4). We found that the tracer signal was diminished in the BnST of BTBR mice, compared with a clear tracer signal in B6 tissue (figure 5D), indicating that degraded neural projections of the PVN to the BnST neurons in BTBR mice. Thirdly, using a retrograde AAV vector encoding mCherry with a selective OXT promoter (retroAAV-pOXT;hM3Dq-mCherry) and anti-OXT staining [37], we also investigated the projections of OXT^PVN^ neurons in both strains and found strain difference in OXT neuron projecting patterns (figure 5E,G). OXT^PVN→BnST^ neurons are distributed widely across the rostral to caudal portions of the PVN in B6 mice, while these OXT^PVN→BnST^ neurons are present in only sparse amounts in BTBR mice (strain: *F*(1,52) = 18.15, *p* = 0.0001, PVN region: *F*(2,52) = 1.63, *p* = 0.205, and interaction: *F*(2,52) = 0.06, *p* = 0.939) (figure 5F). Furthermore, there was no strain difference in the density of OXT^PVN→MeA^ neurons between B6 and BTBR mice (strain: *F*(1,39) = 1.13, *p* = 0.294, PVN region: *F*(2,39) = 5.23, *p* = 0.0098, interaction: *F*(2,39) = 0.83, *p* = 0.442) (figure 5H). The OXT^PVN→MeA^ neurons tend to locate massively in the caudal portion of the PVN in both B6 and BTBR mice.

### 2.4. The oxytocin neurons are involved in the regulation of socio-emotional behaviours

The PVN is a centre brain region for stress responses [32] and anxiety behaviour [20]. While OXT is known to play a modulatory role in anxiety behaviour, the effects of exogeneous OXT are still variable [34] and the brain regions that transmit OXT neuronal signals from the PVN for proper execution of anxiety behaviour have not been identified. Accordingly, BTBR mice display a variable profile of anxiety-like behaviours in the EPM (e.g. increase or decrease) when compared with B6 strains [27,38–40]. In the present study, both male and female BTBR mice exhibited a low anxious profile in the EPM, with increased locomotor activity and enhanced time spent in the open arms compared with control B6 mice (electronic supplementary material, figure S1). Otherwise, sex differences of the behavioural performance in BTBR strain as well as B6 strain were not consistent in this test.

**Figure 6.**
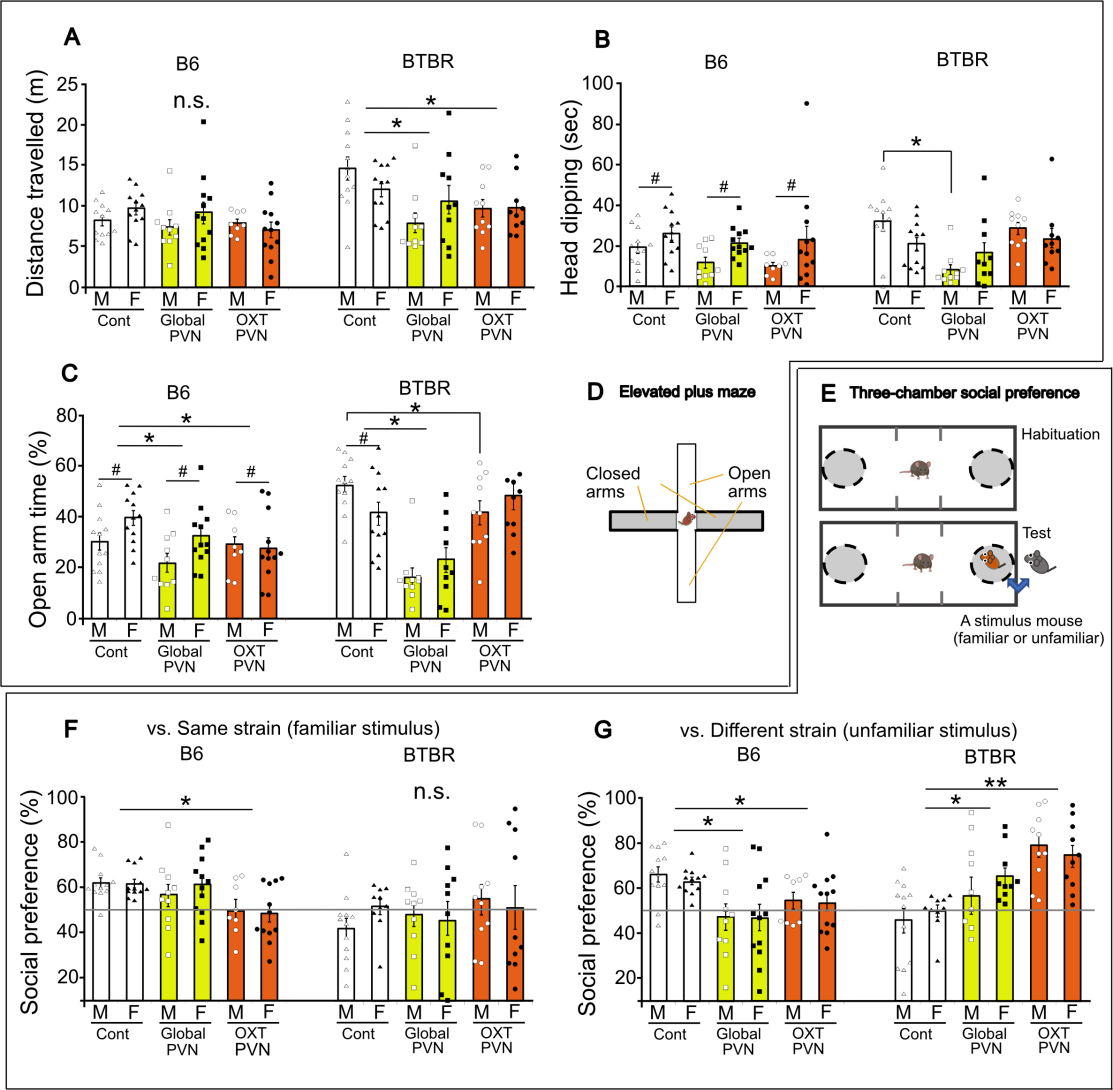
PVN^global^ and OXT^PVN^ neurons are involved in the modulations of anixiety-like behaviour in the elevated plus maze (EPM) and social investigation in the three-chamber test. Chemogenetic activation of entire PVN neurons (PVN^global^) or OXT neurons (OXT^PVN^) before the EPM tests (A–C) or three-chamber test (F,G) in B6 and BTBR mice. Distance travelled (A), locomotor distance, head dipping (B), time dipped head at the arm-edge, and open-arm time (C), time spent in the open arms. (D) EPM. (E) Procedure of the three-chamber test. (F) Social preference ratio, time ratio spent proximity in a social stimulus (familiar cagemate) over an empty bin. (G) Social preference ratio, time ratio spent proximity in a social stimulus (unfamiliar different strain) over an empty bin. *Difference between DREADD manipulation compared with controls, *p* < 0.05. # Difference between sexes, *p* < 0.05. n.s., Not significant.

We applied chemogenetic manipulation to the PVN neurons to determine the OXT cell-type specific function of the PVN circuits. In particular, we bilaterally injected AAV vector with low transduce tropism via a ubiquitin promoter (AAV8-EF1a:hM3Dq; PVN^global^, labelling global PVN neurons) or OXT specific promoter (AAV8-pOXT:hM3Dq; OXT^PVN^) into the PVN (figure 6A). In this scenario, cells expressing hM3(Dq) are categorized as various cell types in the PVN region (PVN^global^) or specifically OXT neurons in the PVN (OXT^PVN^). This manipulation induced selective excitation of targeted neurons by an injection of a DREADD ligand, Compound 21 (C21). Indeed, treatment using C21 alone had no impact on any behaviour performed in following tests (electronic supplementary material, figure S2). Therefore, mice received either AAV injection in the MeA or BnST with vehiclesaline injection as a control.

**Figure 7.**
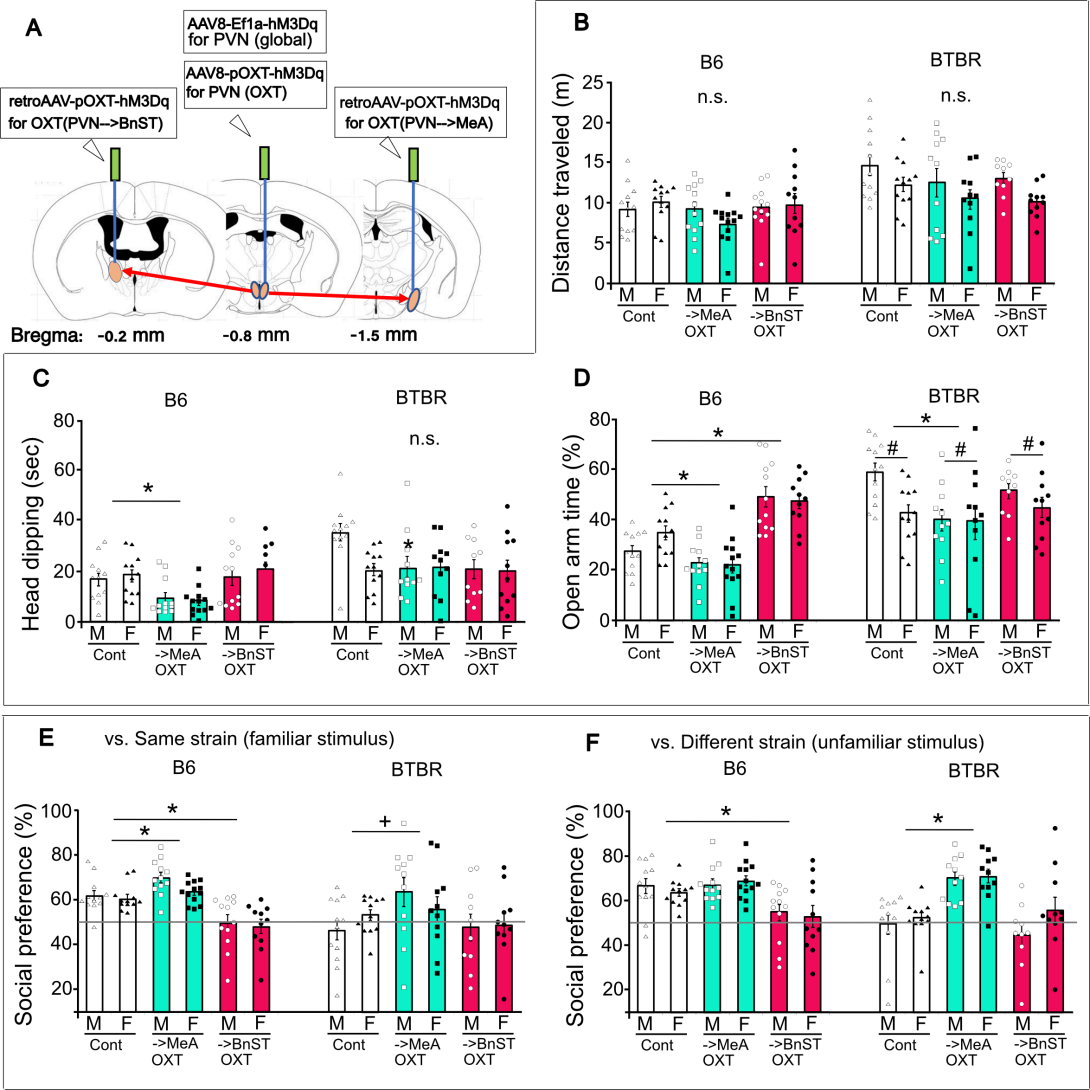
OXT^PVN^ projection-dependent regulations of socio-emotional behaviours in B6 and BTBR mice. (A) The viral strategy transfecting hM3Dq injected into the PVN, MeA, or BnST. These injections were performed for the experiments in figures 4, 5 and 6 (PVN^global^ or OXT^PVN^) or those in figure 7 (OXT^PVN-->MeA^ or OXT^PVN-->BnST^). (B–D) Chemogenetic activation of OXT neurons projecting to MeA (OXT^PVN-->MeA^) or those projecting to BnST (OXT^PVN-->BnST^) before the EPM tests or (E,F) three-chamber test in B6 and BTBR mice. For EPM test, distance travel (B), head dipping (C), and open-arm time (D). For three-chamber test, social preference towards a familiar cagemate (E) and social preference towards an unfamiliar different strain stimulus (F). * Difference between DREADD manipulation compared with controls, *p* < 0.05. # Difference between sexes, *p* < 0.05. n.s., Not significant.

In the EPM (figure 7D), there was no chemogenetic effect or sex difference in the distance travelled of B6 mice (C21: *F*(2,64) = 1.13, *p* = 0.33, sex: *F*(1,64) = 1.07, *p* = 0.305, interaction: *F*(2,64) = 1.06, *p* = 0.353) (figure 7A,B). Female B6 mice showed consistently higher head dipping than male B6 mice regardless of chemogenetic transfection (C21: *F*(2,64) = 1.61, *p* = 0.208, sex: *F*(1,64) = 9.07, *p* = 0.004, interaction: *F*(2,64) = 0.24, *p* = 0.788) (figure 7B, B6). While female B6 mice exhibited a higher open-arm time than male B6 mice, both mice that received chemogenetic excitation of PVN^global^ or OXT^PVN^ neurons (*p*s = 0.032) decreased the open-arm time (C21: *F*(2,64) = 4.99, *p* = 0.01, sex: *F*(1,64) = 6.72, *p* = 0.012, interaction: *F*(2,64) = 1.99, *p* = 0.145) (figure 7C,B). On the other hand, chemogenetic excitation of PVN^global^ (*p* = 0.008) or OXT^PVN^ neurons (*p* = 0.009) decreased the distance travelled in BTBR mice (C21: *F*(2,62) = 6.33, *p* = 0.003, sex: *F*(1,62) = 0.01, *p* = 0.918, interaction: *F*(2,62) = 2.34, *p* = 0.106) (figure 7A, BTBR). Chemogenetic excitation of PVN^global^ neurons decreased the head dipping of male (*p* = 0.002), but not female BTBR mice (*p* = 0.53) (C21: *F*(2,62) = 7.78, *p* = 0.001, sex: *F*(1,62) = 0.61, *p* = 0.437, interaction: *F*(2,64) = 2.92, *p* = 0.062) (figure 7B, BTBR). In addition, chemogenetic excitation of PVN^global^ neurons decreased the open-arm time (*p* = 0.017), while male BTBR mice showed such decreased open-arm time when activated PVN^global^ (*p* = 0.013) but not OXT^PVN^ neurons (*p* = 0.332) (C21: *F*(2,62) = 24.31, *p* < 0.0001, sex: *F*(1,62) = 0.06, *p* = 0.803, interaction: *F*(2,62) = 4.28, *p* = 0.019) (figure 7C, BTBR).

As consonantly presented in various literatures, both male and female BTBR mice demonstrate behavioural traits relevant to autism, including reduced social approaches towards familiar (c.f., same strain cagemate) or unfamiliar (c.f., different strain) social stimuli, compared with B6 controls [30,31,41]. Using chemogenetic manipulation, we uncovered PVN cell-type-dependent regulation of these social behavioural traits in B6 and BTBR mice (figure 7E). When confronted with a same-sex familiar opponent (e.g. B6 stimulus mouse to B6 subjects) (figure 7F), excitation of OXT^PVN^ neurons (*p* = 0.0015), but not those of global PVN neurons (PVN^global^) (*p* = 0.316), decreased social approaches of both male and female B6 mice (C21: F(2,60)= 6.38, *p* = 0.003, sex: F(1,60)= 0.02, *p* = 0.883, interaction: F(2,60)= 0.38, *p* = 0.689). Chemogenetic excitation of either PVN^global^ or OXT^PVN^ neurons had no impact on social preference scores toward a same strain cagemate in BTBR mice (C21: *F*(2,56)= 0.66, *p* = 0.522, sex: *F*(1,56)= 0.24, *p* = 0.626, interaction: *F*(2,56)= 0.94, *p* = 0.397). For the social investigation towards a stimulus of a different strain (figure 7G), chemogenetic excitation of PVN^global^ (*p* = 0.0002) or OXT^PVN^ neurons (*p* = 0.026) decreased the social preference scores in B6 mice (C21: *F*(2,60)= 8.59, *p* = 0.0005, sex: *F*(1,60)= 0.10, *p* = 0.756, interaction: *F*(2,60)= 0.06, *p* = 0.941), while those of PVN^global^ (*p* = 0.027) or OXT^PVN^ neurons (*p* < 0.0001) increased the social preference scores in BTBR mice (C21: *F*(2,56)= 12.8, *p* < 0.0001, sex: *F*(1,56)= 0.42, *p* = 0.522, interaction: *F*(2,56)= 0.74, *p* = 0.48).

### 2.5. Oxytocin projection circuits discretely control socio-emotional behaviour

We propose that the function of OXT neurons on socio-emotional behaviours varies with the projections. We chose to virally manipulate two major OXT projections with retrograde tropism via OXT promoter at the MeA; OXT^PVN→MeA^ or at the BnST; OXT^PVN→BnST^ to examine their socio-emotional function using chemogenetic excitation (figure 6A). In the EPM, there was no impact observed on the distance travelled in both mouse strains under the current DREADD manipulation conditions (OXT^PVN→MeA^ and OXT^PVN→BnST^) (B6; C21: *F*(2,70) = 1.61, *p* = 0.209, sex: *F*(1,70) = 0.03, *p* = 0.869, interaction: *F*(2,70) = 1.39, *p* = 0.255, and BTBR; C21: *F*(2,65) = 1.95, *p* = 0.151, sex: *F*(1,65) = 6.72, *p* = 0.012, interaction: F(2,62) = 0.07, *p* = 0.937) (figure 6B). Chemogenetic excitation of OXT^PVN→MeA^ (*p* = 0.001) but not OXT^PVN→BnST^ neurons (*p* = 0.483) decreased the head-dipping in B6 mice (B6; C21: *F*(2,70) = 10.22, *p* = 0.0001, sex: *F*(1,70) = 0.21, *p* = 0.651, interaction: *F*(2,70) = 0.41, *p* = 0.668), while those chemogenetic excitation did not have impact on the head-dipping in BTBR mice (BTBR; C21: *F*(2,65) = 2.62, *p* = 0.081, sex: *F*(1,65) = 3.14, *p* = 0.081, interaction: *F*(2,65) = 2.74, *p* = 0.073) (figure 6C). Chemogenetic excitation of OXT^PVN→MeA^ neurons decreased the open-arm time in both B6 (*p* = 0.0038) and BTBR mice (*p* = 0.039), while those of OXT^PVN→BnST^ neurons showed strain dependent; increased the open-arm time in B6 mice (*p* < 0.0001) and had no effect on it in BTBR mice (*p* = 0.543) (B6; C21: *F*(2,70) = 38.67, *p* < 0.0001, sex: *F*(1,70) = 0.48, *p* = 0.492, interaction: *F*(2,70) = 1.37, *p* = 0.261, and BTBR; C21: *F*(2,65) = 3.86, *p* = 0.027, sex: *F*(1,65) = 5.07, *p* = 0.028, interaction: *F*(2,65) = 1.71, *p* = 0.189) (figure 6D). These data suggest that activation of OXT^PVN→MeA^ neurons is consistently anxiogenic in both B6 and BTBR mice, while those of OXT^PVN→BnST^ neurons is anxiolytic in B6 mice but no effect in BTBR mice.

Investigatory behaviours in the three-chamber apparatus are also assessed in B6 and BTBR mice that received chemogenetic manipulation of OXT^PVN→BnST^ and OXT^PVN→MeA^ neurons. An excitation of OXT^PVN→BnST^ neurons (*p* < 0.0001) reduced, but those of OXT^PVN→MeA^ neurons (*p* = 0.035) increased social preference scores to a same strain stimulus in B6 mice (B6; C21: *F*(2,66)= 27.57, *p* < 0.0001, sex: *F*(1,66)= 2.59, *p* = 0.113, interaction: *F*(2,66)= 0.75, *p* = 0.476) (figure 6E, B6). The chemogenetic excitation of OXT^PVN→MeA^ neurons tended to increase the social preference scores to a same strain cagemate and those of OXT^PVN→BnST^ neurons did not impact on the scores in BTBR mice (BTBR; C21: *F*(2,61)= 2.92, *p* = 0.062, sex: *F*(1,61)= 0.008, *p* = 0.928, interaction: *F*(2,61)= 1.11, *p* = 0.335) (figure 6E, BTBR). In addition, chemogenetic excitation of OXT^PVN→BnST^ neurons (*p* = 0.0009) but not those of OXT^PVN→MeA^ neurons (*p* = 0.317) tended to decrease the social preference scores to a different, unfamiliar stimulus in B6 mice (B6; C21: F(2,66)= 11.4, *p* = 0.0001, sex: *F*(1,66)= 0.16, *p* = 0.694, interaction, *F*(2,66)= 0.26, *p* = 0.77) (figure 6F, B6). Conversely, the chemogenetic excitation of OXT^PVN→MeA^ (*p* < 0.0001), but not of OXT^PVN→BnST^ neurons (*p* = 0.973) enhanced the investigation scores in BTBR mice (BTBR; C21: *F*(2,61)= 16.76, *p* < 0.0001, sex: *F*(1,61)= 2.52, *p* = 0.117, interaction: *F*(2,61) = 0.92, *p* = 0.406) (figure 6F, BTBR).

These chemogenetic manipulations demonstrate OXT projection dependent processing of social investigation in B6 and BTBR mice, and discretely ineffective activation of OXT^PVN→BnST^ neurons in BTBR mice. Thus, chemogenetic activation of OXT^PVN→MeA^ neurons consistently enhanced the social preference scores in both B6 and BTBR mice, while those of OXT^PVN→BnST^ neurons induced decreased social preference in B6 mice, but no effect in BTBR mice.

## 3. Discussion

### 3.1. Oxytocin circuit-dependent regulation of socio-emotional behaviour

The hypothalamic neuronal network, including OXT neurons in the PVN responds to the dynamic control of approach/avoidance behaviours in social and non-social contexts in a circuit-dependent manner [37,42]. We provided strong evidence using B6 mice, as a standard strain, that the OXT^PVN→MeA^ circuit activates social investigatory approaches, along with the anxiogenic profile, while the OXT^PVN→BnST^ circuit suppresses social approaches, accompanied by an anxiolytic response. These findings are consistent with previous findings of OXT function in social situations: the OXT^PVN→MeA^ neurons are responsive to facilitate social approaches [16,17], while the OXT^PVN→BnST^ neurons promote social avoidance in female California mice [19] and reduce social approaches to stressed mice [18]. In the current study, social encounters with unfamiliar conspecifics induced c-Fos, neural activity marker, across the rostral-to-caudal portions of the PVN and its projecting regions, including the BnST (only the anterior portion), MeA, and LS of B6 mice, in which the induction of *OXT* and *OXT receptor* genes was also evident, except in the septum, where *AVPR1a* was upregulated. This neural activity mapping in response to a social encounter indicates a role for OXT circuits corresponding to social processing. Both social encounters and chemogenetic excitation of OXT^PVN^ neurons concurrently activated several OXT-projecting regions, including BnST and MeA, and upregulated OXT receptor genes in the terminal regions. However, chemogenetic excitation of OXT^PVN^ neurons did not induce *c-fos* mRNA expression in these projecting regions but decreased social investigation in the three-chamber test and the time spent in the open arms in the EPM in B6 mice.

Weak *c-fos* gene induction in these projecting regions by chemogenetic excitation of OXT^PVN^ neurons could be ascribed to the cell-type-specific locality of chemogenetically activated projections that do not sufficiently induce global neural activation in the projecting nuclei. Nevertheless, excitation of OXT^PVN^ neurons induces behavioural changes along with OXT receptor gene upregulation in the projecting regions. When the behaviour of B6 mice with chemogenetic excitation of OXT^PVN^ neurons was compared with those with projection-specific excitations, chemogenetic excitation of both OXT circuits simultaneously (i.e. OXT^PVN^ excitation) induced decreased social investigation in the three-chamber test and open arm time in the EPM. Accordingly, the excitation of OXT^PVN→BnST^ neurons decreased social investigation and then increased the time spent in the open arm in the EPM, while the excitation of OXT^PVN→MeA^ neurons increased social investigation and decreased the time spent in the open arms. These comparisons of projection-specific manipulations may implicate that OXT^PVN→BnST^ signalling is more effective in social situations when both circuits are simultaneously and exogenously activated (i.e. OXT^PVN^ activation); conversely, OXT^PVN→MeA^ signalling is dominant in a non-social (i.e. EPM) situation. Considering the nature of the functional network properties of OXT, neural projection-dependent activities are required for the execution of coherent functional outputs of the OXT neural network, and chemogenetic excitation of entire OXT^PVN^ neurons without projection-discrete regulation may result in unnatural process of the OXT network.

Both social and object (i.e. non-social) encounters activate investigatory behaviour accompanied by the upregulation of c-Fos in the MeA and BnST in B6 mice. The c-Fos expressing (i.e. activated) OXT^PVN and SON^ neurons were more abundant in social encounters than in object encounters. In addition, OXT^PVN→MeA^ signalling promotes social investigation, while OXT^PVN→BnST^ signalling stimulates non-social (i.e. in EPM) investigation. Therefore, it could be understood as natural (i.e. under non-manipulated condition) OXT^PVN→MeA^ signalling takes priority over those OXT^PVN→BnST^ signalling to control behaviour in a social context, whereas OXT^PVN→BnST^ signalling has a dominant role in the context of a non-social (e.g. possibly a threating stimulus) encounter. A possible key determinant of OXT circuit balance is an inhibitory mediator downstream of the OXT pathway in the BnST. The posterior BnST and the MeA are anatomically characterized as a set of interneural nuclei, known as the extended amygdala [43]. Certain inter-neuronal (possibly GABAergic) connections relayed between the BnST and MeA act to adjust the output balance of neural signals from the investigation of non-social external stimuli (i.e. OXT^PVN→BnST^ signals) to activate the investigation of social stimuli (i.e. OXT^PVN→MeA^ signals). Accordingly, the MeA–posterior BNST circuits projecting to the (dorsal/ventral medial) hypothalamus are involved in the regulation of innate social and predator-defence behaviours [44,45]. Further research is needed to elucidate whether the downstream molecular pathway of OXT circuits maintains the balance of their output within the PVN inter-neuronal connections or across inter-regional connections, such as the MeA-posterior BnST circuits.

### 3.2. Defect of oxytocin circuits accompanied by social deficits in BTBR mice as a mouse model of autism spectrum disorders

OXT neurons play a critical role in processing social approaches and avoidance in a variety of contexts, implying circuit-specific OXT action [12,14]. BTBR mice as an ASD mouse model exhibited enhanced locomotion and longer time in the open arms in the EPM, and diminished social preference in the three-chamber test as compared with standard B6 mice (electronic supplementary material, figure S1). An altered OXT neuronal morphology and activity were also evident in BTBR mice. We provide evidence that the circuit-specific control of OXT in prosocial and anxiety-like behaviours is disrupted in BTBR mice.

BTBR mice exhibit a typical behavioural profile resembling that of humans with ASD, including impaired prosocial interaction and persistent-repetitive behaviours [27,28]. Although the target neuronal circuit responsible for controlling the social and non-social deficits exhibited in animal models of ASD has been investigated for more than a decade, little is known about the circuit mechanisms underlying these behavioural impairments. The present study demonstrated that BTBR mice exhibit decreased densities of OXT neurons in the PVN and decreased ratio of activated (c-Fos co-expressed) OXT^PVN and SON^ neurons in response to a social encounter. A broad defect of c-Fos represented neural activity across OXT-projecting regions, including the PVN, BnST, MeA and LS, in response to a social encounter in BTBR mice. Blunted *OXTR* upregulation in the MeA and BnST in response to a social encounter has also been demonstrated in BTBR mice, indicating a deficiency in OXT-OXTR signals in specific circuits of BTBR mice. Furthermore, both the PVN global and OXT-specific projections to the BnST, which appeared abundantly in B6 mice, were defective in BTBR mice. These malformations of the OXT circuit are linked to blunted neuronal activity patterns across the PVN and its projecting regions. Accordingly, both chemogenetic excitation of entire OXT^PVN^ neurons and selective OXT^PVN→MeA^ neurons can reverse social approach in the three-chamber test and decreased the time in the open arms in the EPM in BTBR mice, whereas excitation of OXT^PVN→BnST^ neurons had no impact on the behaviour of BTBR mice. While there is a certain dissociation between protein levels (e.g. c-Fos IHC) and mRNA levels (e.g. *c-fos* qPCR) in response to a social stimulus exposure, the OXT^PVN→BnST^ circuit, including global PVN projections to the BnST, was functionally defective, but the OXT^PVN→MeA^ circuit was still intact in BTBR mice.

We recently reported circuit dysfunction in BTBR mice characterized by compromised circuit from the posterior BnST to the lateral habenula, possibly via attenuation of vasopressinergic signals/projections, contributing to a lack of coordinative marking responses of social scent signalling in BTBR mice in a social context [31]. Our present data extend the OXT circuit defects in BTBR mice to the control of prosocial and anxiety-like behaviours. Downregulation of AVP 1 a receptors in the BnST was observed in both B6 and BTBR mice when OXT^PVN^ neurons were chemogenetically activated. Similarly, upregulation of *AVPR1a* was induced in the septum in both strains in response to social encounters in current study. Such crosstalk between OXT^PVN^ and AVP^BnST^ in behavioural processes and its deficiencies are key components in understanding how the sequential processes of social behaviour are regulated and disturbed [46]. BTBR mice exhibit agenesis of several brain structures, including the absence of the corpus callosum, atrophy of the hippocampus and glial cell overexpression in the cingulate cortex [31,47,48]. MRI analysis revealed decreased cortical and thalamic grey matter volumes, along with a reduction in cortical thickness in BTBR mice [49]. This indicates that these morphological alterations occur in the developing brain network of BTBR mice, resulting in subsided OXT and AVP signals along with coordinative circuit dysfunctions that are required to express appropriate approach/avoidance behaviour and subsequent processes in social and non-social contexts.

The PVN is a multitransmitter nucleus that controls various biological functions [20]. Corticotropin-releasing hormone, CRH, a PVN hormone regulating hormonal stress reactions, activates self-grooming and defensive behaviours [50–52]. Virally induced selective manipulation of AVP neurons in the PVN can modify anxiety-like behaviour and self-grooming in both sexes and mediate social investigation only in female mice [53,54]. This suggests that stress-related, OXT-independent mechanisms are involved in controlling the compulsive and defensive components of behaviour in the PVN; thus, present chemogenetic excitation of global PVN neurons was anxiogenic in both strains. The BTBR mouse model is associated with deficits in social communication and pronounced engagement in repetitive behaviour [27,55,56]. Excessive self-grooming, persistent digging, and burying of floor bedding materials are considered to represent the later pathological states [57–59]. The characteristics of BTBR mice suggest that the variant circuitry controlling the exploratory and defensive responses underlying their atypical behaviour needs to be further elucidated.

It should be noted that hypothalamic OXT neurons express the vesicular glutamate transporter 2 (*VGlut2*) gene, indicating that they can release glutamate alongside OXT [41,60]. Chemogenetic excitation via hM3 receptors initiates the Gq signalling pathway thus activates both glutamate and OXT release [14,61]. Although the use of chemogenetic manipulation has a technical limitation in specifying selective transmission, the co-release of glutamate and OXT appears to sustain a range of natural neural signalling in regulating socio-emotional behaviours [46]. Another limitation that needs to be addressed is that the AAV-*OXT* promoter construct used in the present study exhibited narrow selectivity (approx. 80%) for transduction into OXT neurons; thus, other cell types of PVN neurons also expressed mCherry (i.e. hM3Dq). Although multiple measurements of OXT and its receptors can provide sufficient justification for chemogenetic manipulation, they are not available for OXT cre-driver BTBR mice. Similarly, chemogenetic excitation by hM3Dq should be used in combination with chemogenetic inhibition by hM4Di to accurately examine neural circuits of interest using bidirectional manipulation, if available.

## 4. Conclusions

The present data demonstrate a circuit-dependent role of OXT^PVN^ neurons in controlling social and emotional behaviours and illustrate that the coordinative regulation of two discrete OXT neurons, the OXT^PVN→BnST^ and OXT^PVN→MeA^ circuits, underlies the control of these behaviours. OXT^PVN→MeA^ neurons are responsible for the expression of anxiogenic behaviour and social investigation in both B6 and BTBR mice, whereas OXT^PVN→BnST^ neurons determine the induction of anxiolytic behaviour and a reduction in social investigation (i.e. enhanced avoidance). A functional defect of the OXT^PVN→BnST^ signal coincided with prosocial behavioural deficits in the BTBR mouse model of ASD. This study provides valuable information on the coordinative role of OXT circuits and establishes a foundation for further investigations to delineate the circuit mechanisms of OXT signalling that impact prosocial processes and social deficits relevant to ASD symptoms [62].

## 5. Methods

### 5.1. Animals

BTBR T+Itpr3 tf/J (BTBR) mice (stock no. 002282) and C57BL/6J (B6) mice (stock no. 000664) of both sexes were originally purchased from The Jackson Laboratory (Bar Harbor, ME, USA), were used as subject and stimulus mice. They were bred and maintained in the colony room of a facility at the University of the Ryukyus Faculty of Medicine, Japan. The mice were housed in groups of three or four in standard shoebox cages (28 × 22.5 × 14 cm height) with water and food provided ad libitum. The vivarium was maintained at 50% humidity and 23±1°C, with a 12 h light/dark cycle (lights on at 08.00). All procedures including animal care and use were performed in line with the National Institutes of Health Guide for the Care and Use of Laboratory Animals that comply with the ARRIVE guidelines and approved by the Institutional Laboratory Animal Care and Use Committee at the University of the Ryukyus Graduate School of Medicine.

### 5.2. Experimental design

We firstly measured c-Fos neural activity in the PVN sub-portions (rostral to caudal) and related projecting regions of B6 (*n* = 22) and BTBR mice (*n* = 21) in response to a novel social or object encounter, and OXT neurons co-expressed with c-Fos in the PVN and SON regions in B6 (*n* = 20) and BTBR mice (*n* = 22) in response to a novel social or object encounter (figure 1). Second, mRNA expressions of targeted genes in social processing brain regions following a social encounter or chemogenetic activation of OXT^PVN^ neurons were evaluated in B6 (*n* = 32) and BTBR mice (*n* = 30). We then conducted two behavioural tests to assess social and non-social profiles relevant to autism-like behaviours in B6 and BTBR mice. Our behaviour tests consisted of anxiety/locomotor task (the elevated plus maze (EPM)) and social behaviour task (the modified three-chamber test). These subject mice including male and female received a bilateral AAV injection for DREADD manipulation and assigned into two groups of AAV conditions (Group 1: vehicle; B6, *n* = 24, BTBR, *n* = 26, PVN^global^; B6, *n* = 22, BTBR, *n* = 20, and OXT^PVN^; B6, *n* = 24, BTBR *n* = 24, and Group 2: vehicle; B6, *n* = 24, BTBR, *n* = 24, OXT^PVN→MeA^; B6, *n* = 25, BTBR, *n* = 22 and OXT^PVN→BnST^; B6, *n* = 23, BTBR, *n* = 21). Due to the misplacement of AAV injection, the following numbers of mice were excluded from data analysis (PVN^global^; B6, *n* = 2, BTBR, *n* = 3, OXT^PVN^; B6, *n* = 3, BTBR *n* = 3, OXT^PVN→MeA^; B6, *n* = 4, BTBR, *n* = 4 and OXT^PVN→BnST^; B6, *n* = 2, BTBR, *n* = 3). Testing was performed in tandem with small groups of mice consisting of three-to-four cohorts of animals at different times. Following a completion of behavioural testing, AAV transduction in the targeted brain sites was histologically verified by microscopy with transduced fluorescence and immunohistochemical staining of OXT neurons.

### 5.3. Adeno-associated viruses

AAV8-EF1a:hM3Dq-cgfTag-RFP and AAV8-EF1a:mCherry was donated by the Fukushima Medical University Virus Vector Core; plasmid package pAAV-pOXT:hM3Dq-mCherry-WPRE was purchased from Addgene (no. 70717) [63], and packaged in serotype AAV8 and retro-AAV2 at the Gunma University Virus Vector Core. For all mice, we allowed for a minimum of 3 weeks of viral expression before conducting any experiments described in the current study.

### 5.4. Stereotaxic surgeries

Adult mice (B6 or BTBR) were deeply anaesthetized with a mixture of anaesthetic compounds of medetomidine (0.3 mg kg^-1^ body weight, b.w.), midazolam (4.0 mg kg^-1^ b.w.) and butorphanol (5.0 mg kg^-1^ b.w.) [64] and placed on a stereotaxic apparatus (Stoelting Co., IL, USA). The injection was carried out with a microinjector (Nanolitre 2020 injector, WPI, FL, USA) at a speed of approximately 0.05 μl min^-1^ with a grass capillary needle (inner diameter > 50 nm). All injections were done bilaterally. The following coordinates were used: PVN, −0.8 mm anterior–posterior (AP), ± 0.15 mm medial–lateral (ML), −4.8 mm dorsal–ventral (DV); MeA, −1.60 mm AP, ± 1.85 mm ML, −5.3 mm DV; and BnST, −0.20 mm AP, ± 0.80 mm ML, −4.20 mm DV. For the virus injection, 50 nl of viral solution per each site was injected into the bilateral PVN and 100 nl of viral solution per each site was injected into the bilateral MeA and BnST. The titres of stock AAV viruses were adjusted to 1.0 × 10^13^ particles ml^-1^ before injections. After each viral injection, the glass micropipette was left in place for 5 min and then slowly withdrawn. The skin was sutured closed. Mice fully recovered under a heat pad before returning to their home cage. Animals were allowed to recover for 2 days in a singly housed cage and used for experiments 3 weeks later.

### 5.5. Social or novel object exposure for targeted brain activity mapping

Subject mice (B6 or BTBR) were exposed to either a social stimulus (novel same-sex mouse) or novel object stimulus (wooden egg or small garden pot) by introducing the stimulus to a clean cage with sawdust bedding. The stimulus was placed in the test cage for 10 min and then removed. The subject mice remained in the test cage for 1.5 h and were then quickly anaesthetized by expiratory isoflurane and sacrificed by transcardial perfusion using PBS and 4% PFA. Brains were extracted and stored in 4% PFA for 24  h, and cryoprotected in 30% sucrose in 1× PBS for 2−3 d. Brains were then embedded in OCT compound and frozen at – 80 °C until further processed.

### 5.6. Immunohistochemistry

Coronal sections (40 μm) of the selected brain regions were prepared using a cryostat (Leica CM1860, Germany). For c-Fos and other immunohistochemistry, free-floating sections were immersed in a blocking solution (10% block ACE (BUF029A, Bio-Rad), 0.4% Triton X-100 in PBS) for 2 h at room temperature. Sections were incubated overnight at 4◦C with the primary antibody: rabbit polyclonal anti-c-Fos (1 : 1000 in blocking solution; cat. no. PC05, EMD Millipore) or 1 : 1000 anti-c-Fos antibody (cat. no. 226003, SySy), rabbit polyclonal anti-OXT (1 : 2000 in blocking solution; cat. no. 20068, lot no. 1607001, Immunostar, WC, USA) or mouse monoclonal anti-NeuN (1 : 1000 in blocking solution; MAB377, EMD Millipore, MA, USA). Sections were then incubated with a fluorophore (1 : 500, Alexa Fluor 488 or 594, Jackson ImmunoResearch, PA, USA) goat anti-rabbit secondary antibody for 2 h at room temperature with agitation.

c-Fos protein fluorescence was visualized on a Nikon Eclipse Ni-E fluorescence microscope with a 10× objective lens. The numbers of c-Fos+cells were determined using ImageJ 1.53. We generated a threshold and voxel number to isolate c-Fos nuclear signal by randomly selecting four images from each condition (12 total) and optimized a threshold and voxel number that yielded an accurate representation of c-Fos positive nuclei in each image. Those numbers were applied to the whole dataset. The density data for c-Fos+cells was calculated as the average of 3−4 sections data (count per 100 μm^2^ section) taken from each region.

### 5.7. Microdissection for quantitative polymerase chain reaction mRNA analysis

Subject mice received bilateral PVN injections with AAV8-pOXT:hM3Dq. Three weeks after the surgery, mice were assigned either three conditions; control, mice received i.p. saline vehicle injection and remained in their home cage for 60 min; social, mice received i.p. saline vehicle injection and 30 min later they are moved to a clear cage and confronted with a glid bin containing a same sex stimulus mouse of different strain for 10 min (see figure 2H So group), and then, the stimulus mouse (and bin) were removed and the subject mice were remained for 30 min; and DREADD, mice received C21 (1 mg/kg, i.p.) and remained in their home cage for 60 min. Following either treatment, mice were anaesthetized with isoflurane, brains were removed and immediately sectioned in the coronal plane at 1 mm on an ice-cold metal brain slicer (Brain matrice, Agntho’s AB, Sweden), and slices were transferred into RNAlater solution (Sigma-Aldrich) and stored at 4°C. The selected brain regions, including PVN, MeA, BnST and septum, were dissected from brain slices under ×10 microscopy using mouse brain atlas [65] as a reference, within 3 days after immersed in RNAlater. These dissected tissues were immersed in 400 µl of TRIzol reagent (Life Technologies Corporation, CA, USA) and stored at −80 °C until further processing for total RNA extraction.

### 5.8. RNA extraction and cDNA Synthesis

Total RNA was extracted using 400 µl of TRIzol reagent and 80 µl chloroform, precipitated with 200 µl of 2-propanol (Nacalai tesque, Kyoto, Japan). Pellets were washed twice with 75% ethanol, air-dried and dissolved in ultra-pure water. RNA concentrations were calculated using NanoDrop™ One (ThermoFisher Scientific). A total of 250 ng of total RNA were loaded in each cDNA synthesis reaction. cDNA synthesis was conducted using the TC1000-G thermal cycler (DLAB Scientific Co., Ltd, Beijing, China) in a final volume of 20 µl. Each reaction contained 250 ng of RNA diluted in a volume of 10 µl, to which we added 10 µl of 2X Reverse Transcription Master Mix (High Capacity cDNA Reverse Transcription Kit with RNase Inhibitor, Applied Biosystems, CA, USA). Samples were incubated at 25°C for 10 min, 37°C for 120 min, followed by 85°C for 5 min. Finally, cDNA samples were stored at −20 °C until use.

### 5.9. Real-time quantitative polymerase chain reaction

Real-time qPCR analyses were carried out as previously reported [16,41], with minor modifications. For each gene of interest, qPCRs were performed in a final volume of 10 μl, which comprised 1 μl cDNA, 3.5 μl of ultrapure water, 5 μl of GoTaq master mix (Promega, WI, USA) and 0.2 μl of the corresponding forward and reverse primers (10 μM, Greiner Bio-One Co., Tokyo, Japan) to obtain a final primer concentration of 200 nM. The primers are described in electronic supplementary material, table S1. Reaction mixtures were loaded in eight-well PCR Tube Strips with Ergonomic caps (AlphaGem Bio, CA, USA), and genes of interest were tested using the StepOnePlus Real-Time PCR System (Applied Biosystems, MA, USA). Instrument settings were as follows: (i) 95°C for 2 min, (ii) 95°C for 3 s, (iii) 60°C for 30 s, (iv) plate read, (v) repeat step 2 to 4 for 40 cycles, followed by a dissociation stage. Determination of gene transcript in each sample was obtained by the ΔΔCt method using β-actin as a reference gene. To examine changes in expression, we analysed the mean fold change values of each sample compared to a sample of B6 control as a targeted control. PCR product specificity was evaluated by melting curve analysis, with each gene showing a single peak (data not shown).

### 5.10. Behavioural tests

All behavioural testing was conducted during the light cycle (at 12.00−19.00) in separate experimental rooms when the subject and stimulus mice were ready to use. We conducted three types of tests to explore several aspects of behavioural features including the locomotion and anxiety (e.g. the EPM), social approach (e.g. modified three-chamber test), and defence behaviour (e.g. odour exposure defence test), in fixed-order of testing. The inter-test interval was 1 week, to avoid any residual effect of C21 (Hello Bio, UK) treatment. Thus, we set up five groups of DREADDs stimulation; vehicle, mice were given AAV (either AAV8-EF1a: hM3Dq or AAV8 or retro-AAV2-pOXT: hM3Dq) injection either PVN, MeA or BnST, and received saline injection when tested; non-specific PVN activation (PVN^global^), mice were injected with a AAV8-EF1a: hM3Dq into the PVN, and received a C21 (1 mg/kg, i.p.) treatment before the tests; OXT specific PVN activation (OXT^PVN^), mice were injected with a AAV8-pOXT: hM3Dq into the PVN, and given a C21 treatment before testing; OXT specific PVN to MeA circuit activation (OXT^PVN→MeA^), mice bilaterally received an injection of retroAAV-pOXT: hM3Dq into the MeA, and were injected with a C21 when tested; OXT specific PVN to BnST circuit activation (OXT^PVN→BnST^), mice were bilaterally given an injection of retroAAV-pOXT: hM3Dq into the posterior BnST, and received a C21 treatment when tested.

### 5.11. Elevated plus maze

The EPM consists of two open arms and two closed arms situated opposite each other and separated by a 6 cm square centre platform (figure 6D)[66]. Each runway is 6 cm wide and 30 cm long. The open arms have lips that are 0.5 cm high, and the closed arms are surrounded on three sides by 20 cm walls. The mouse was placed into the intersection of the open and closed arms and allowed to move freely for 5 min. The distance travelled and the percentages of time spent in open arms were recorded using ANY-maze software (Stoelting Co. IL, USA). The maze was cleaned between each animal.

### 5.12. Modified three-chamber test

The apparatus used for the social preference test has been previously described [53,67,68]. The chamber consisted of an open-topped box divided into three sections. Two inverted grid bins were placed on each compartment (figure 6E).

After 40 min of acclimatization to the environment of the experimental room, the mice were placed in the centre box and the mice were allowed to explore freely for 7 min. The test sessions consisted of three phases: the first, the habituation phase, and the second and third, social exposure phases. During the habituation phase, each side chamber contained an empty bin. During the social exposure phase, a mouse (a same-sex BTBR or B6 stimulus mouse) was enclosed in one of the bins in a side chamber. The location and order of the introduction of the stimulus mouse were counterbalanced. After the test sessions, the subject mice were returned to their home cages and the apparatus was cleaned with 15% ethanol and dried with a paper towel. The behaviours of the subject mice were recorded on video and analysed for investigatory behaviours when oriented to the grid bins using ANY-maze software (Stoelting Co., IL, USA). The time that each mouse spent in the proximity of each bin (distance of animal head to bin edge: less than 3 cm) was measured. Preference (approach) was calculated as the percentage of time spent in the proximity of the bins containing a stimulus mouse from the total time spent in both bin proximities in a 7 min test.

### 5.13. Statistical analysis

All data are expressed as the mean ± standard error of the mean, and individual data dot-plotted. For the analysis of c-Fos expression, data were analysed by one-way analysis of variance (ANOVA) with the between-subject factor of the condition (Co, homecage control, So, social encounter, and Ob, object exposure). For the percentage data of OXT+cells co-expressed with c-Fos, a two-way ANOVA with stimulus (Ob versus So) and sex as between-subject factors, was conducted. For the behavioural data in exposure conditions, two-way ANOVA with the between-subject factors of the sex and AAV (group 1: Cont, PVN^Global^, versus OXT^PVN^, and group 2: Cont, OXT^PVN→MeA^, versus OXT^PVN→BnST^) was utilized. Densities of OXT+cells in the PVN were analysed by a two-way ANOVA with strain (B6 versus BTBR) and sex. The percentage of merged cells were analysed by a two-way ANOVA with strain (B6 versus BTBR) and PVN portions (rostral, middle and caudal). Finally, mRNA expression data were analysed by two-way ANOVA with strain (B6 versus BTBR) and stimulus condition (C, control, S, social stimuli, and D, DREADD with OXT neurons into the PVN). For all ANOVA analyses, we used the Bonferroni correction for *post hoc* comparisons when necessary. For all tests, differences were considered statistically significant at *p* < 0.05. All statistical analyses were performed using R Statistical Software version 4.1.2. with package Psych version 2.1.3.

## Data Availability

The datasets collected and analysed in this study, including staining counting data, mRNA expression data, and behavioral data, are available from Figshare [69]. Supplementary material is available online [70].
